# Central obesity associates with renal hyperfiltration in the non-diabetic general population: a cross-sectional study

**DOI:** 10.1186/s12882-016-0386-4

**Published:** 2016-11-10

**Authors:** Vidar Tor Nyborg Stefansson, Jørgen Schei, Trond Geir Jenssen, Toralf Melsom, Bjørn Odvar Eriksen

**Affiliations:** 1 Metabolic and Renal Research Group, Faculty of Health Sciences, UiT The Arctic University of Norway, Tromsø, Norway; 2 Section of Nephrology, University Hospital of North Norway, Tromsø, Norway; 3 Department of Organ Transplantation, Oslo University Hospital, Oslo, Norway

**Keywords:** Body mass index, Chronic kidney disease, Glomerular filtration rate, Glomerular hyperfiltration, Waist circumference, Waist-hip ratio

## Abstract

**Background:**

Obesity is a risk factor for end-stage renal disease. Renal hyperfiltration, defined as an abnormally high glomerular filtration rate (GFR), is a link in the causal chain between diabetes and chronic kidney disease. Whether obesity is associated with hyperfiltration in the non-diabetic general population, remains unresolved due to a lack of consensus regarding the definition of hyperfiltration and the limited precision of high-range GFR estimations with creatinine and/or cystatin C.

**Methods:**

1555 middle-aged participants without diabetes, renal or cardiovascular disease were enrolled from the general population in the Renal Iohexol Clearance Survey from the 6th Tromsø Study (RENIS-T6) between 2007 and 2009. Obesity was assessed using the body mass index (BMI), waist circumference (WC) and the waist-hip ratio (WHR). GFR was measured by iohexol clearance. Dichotomous variables for hyperfiltration were based on two alternative definitions using unadjusted GFR (mL/min) above the 90th percentile. The 90th percentile was age-, sex- and height-specific in one definition and age-, sex-, height- and weight-specific in the other.

**Results:**

In multivariable adjusted logistic regression models, only WHR was consistently associated with hyperfiltration based on both definitions. For the definition based on the age-, sex-, height- and weight-specific 90th percentile, the association with the WHR (odds ratios (95 % confidence intervals)) for hyperfiltration was 1.48 (1.08–2.02) per 0.10 WHR increase.

**Conclusions:**

Central obesity is associated with hyperfiltration in the general population. The WHR may serve as a better indicator of the renal effects of obesity than BMI or WC.

**Electronic supplementary material:**

The online version of this article (doi:10.1186/s12882-016-0386-4) contains supplementary material, which is available to authorized users.

## Background

The prevalence of obesity, defined as a body mass index (BMI) ≥ 30 kg/m^2^, has increased rapidly in high-income nations over the last few decades and is steadily growing in many lower-income countries as well [1]. Obesity is a well-known risk factor for cardiovascular disease, hypertension and diabetes [2, 3]. These diseases are, in turn, well-established risk factors for chronic kidney disease (CKD) and end-stage renal disease (ESRD) [4–7]. However, there is also evidence of a direct causal connection between obesity and ESRD, independent of hypertension and diabetes [8, 9].

Renal hyperfiltration (RHF), or an abnormally high glomerular filtration rate (GFR), has been postulated to represent an early stage in the development of CKD [10], most clearly observed in diabetic nephropathy [11]. RHF is also associated with several established CKD risk factors, including hypertension [12, 13] and smoking [14, 15]. A large longitudinal study by Park et al. of 43,503 Korean health screening participants found that a RHF definition based on eGFR was associated with all-cause mortality, even when adjusted for age, sex, muscle mass, diabetes and hypertension [16]. Although several studies have been conducted on the relationship between obesity and RHF [17–25], it remains unclear whether these two conditions are also associated in the general non-diabetic population. The most important reason has been that there is currently no consensus on the definition of the term “hyperfiltration”. Most investigators who defined RHF in their studies used a single GFR cut-off point and adjusted their definition for no other variable than body surface area (BSA) [26]. Although there is no generally accepted way of defining RHF, it has been suggested that the definition should use age and sex-specific cut-offs and also some measure of correction for body size [26, 27].

Another methodological problem has been that previous epidemiological studies used GFR estimates based on creatinine and cystatin C, rather than GFR measurements [21–25]. Estimated GFR is inaccurate for high-range GFR [28–30] and can be confounded by associations with non-GFR-related factors [31, 32]. Studies on obesity and RHF using measured GFR (mGFR) have been limited by small sample sizes [17–19] and the lack of adjustment for confounding variables [20].

In the Renal Iohexol Clearance Survey in Tromsø 6 (RENIS-T6), we measured GFR with iohexol clearance in 1627 middle-aged subjects from the general population. The aim of the present study was to examine the relationship between obesity and two alternative definitions of RHF.

## Methods

### Subjects

**Fig. 1:**
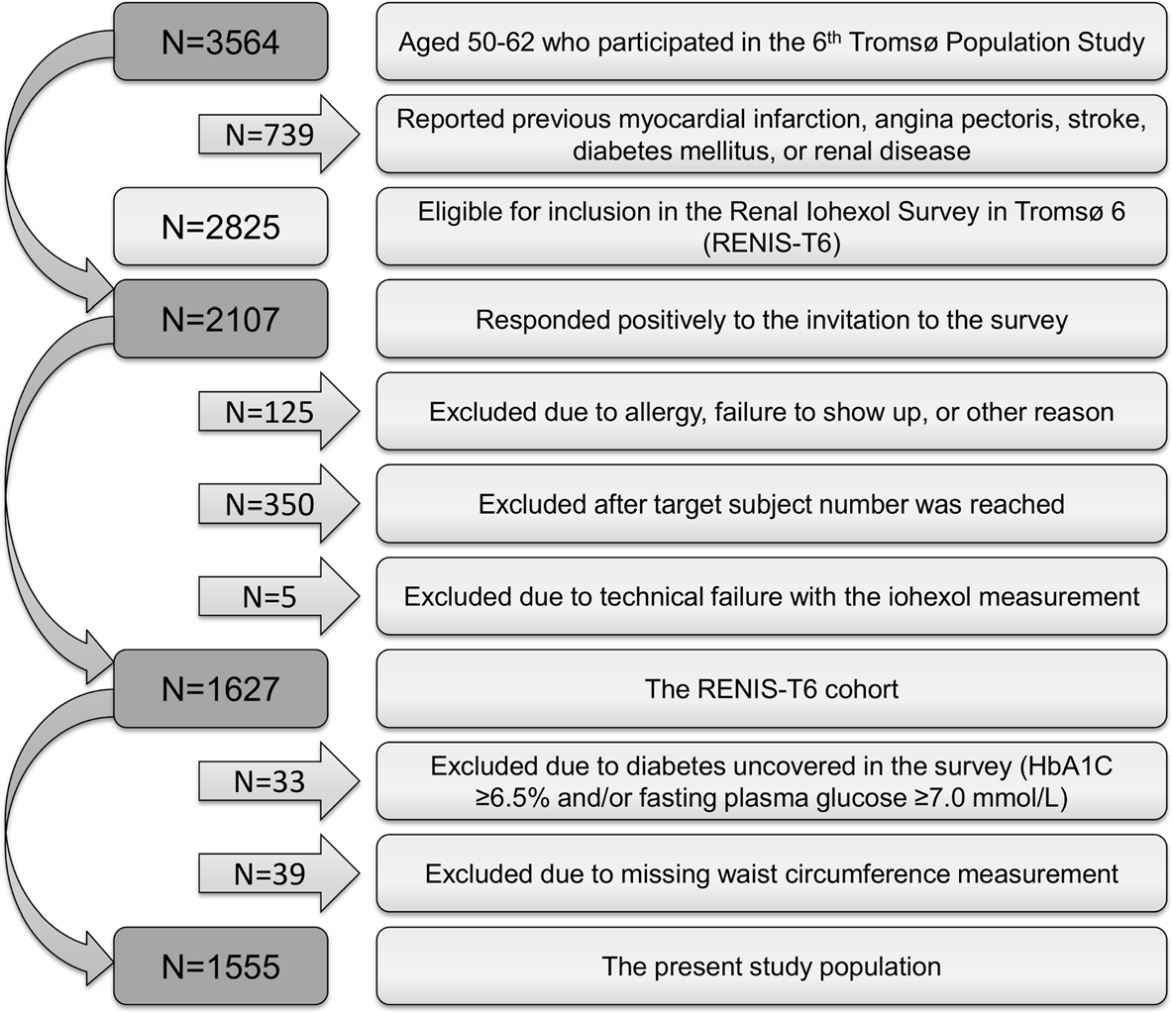
Inclusion of subjects in the RENIS-T6 cohort and the present study population

RENIS-T6 was conducted from 2007 to 2009 as a substudy of the sixth Tromsø Study (Tromsø 6). The RENIS-T6 cohort consisted of a representative sample of 1627 persons from the general population of Tromsø who were between 50 to 62 years of age and without self-reported diabetes mellitus, cardiovascular or kidney disease (Fig. 1); the cohort has previously been described in detail [28].

Subjects were excluded from the present study if they had previously undiagnosed diabetes mellitus (hemoglobin A1c ≥ 6.5 % and/or fasting plasma glucose ≥ 7.0 mmol/L) or if they lacked waist or hip circumference measurements.

Smoking status was ascertained as part of a detailed questionnaire in the Tromsø 6 study. Previous smokers were grouped with non-smokers for the purposes of this study. Medication use was ascertained separately in the RENIS-T6 study. Antihypertensive medication use was categorized into six categories: beta-blockers, calcium channel blockers, diuretics, angiotensin converting enzyme-inhibitors, angiotensin-II receptor blockers, and other antihypertensive medications.

### Iohexol-clearance measurements

Examination of the subjects started between 08:00 and 10:00 AM. All subjects had fasted and abstained from smoking since midnight, and they were instructed not to use non-steroid anti-inflammatory drugs or eat large quantities of meat during the preceding 48 h. Subjects were instructed to drink two to three glasses of water before arrival. A Teflon catheter was placed in an antecubital vein and was flushed with 30 mL of isotonic saline. Five milliliters of iohexol (Omnipaque, 300 mg/mL; Amersham Health) was injected, and the syringe was weighed before and after injection. GFR was measured as the single-sample plasma clearance of iohexol, a method that has been validated against gold standard methods [33], and analyzed using high-precision liquid chromatography as described by Nilsson-Ehle [34]. The analytical variation coefficient for the study period was 3.0 %. Jacobsson’s method was used to calculate the GFR [35]. Further procedural details have been described previously [28].

### Laboratory measurements

Glucose, low-density and high-density lipoprotein cholesterol, and triglycerides were measured on a Modular P800 (Roche Diagnostics, Basel, Switzerland). The insulin concentration was measured with an enzyme-linked immunosorbent assay kit (DRG Instruments, Marburg, Germany), with intra- and interassay coefficients of variation of 4.7 % and 6.3 %, respectively. Insulin resistance was expressed by the homeostasis model assessment (HOMA-IR), calculated by multiplying fasting glucose (mmol/L) by fasting insulin (mU/L) and dividing the result by 22.5 [36].

### Blood pressure measurement

Office blood pressure (BP) was measured at the study site using an automated device (model UA799; A&D, Tokyo, Japan) after 2 min of rest. Daytime ambulatory BP was measured using weighted daytime (10:00–22:00) averages of BP measured at 20-min intervals. Further details of the BP measurements have been described previously [37].

### Body measurements

Waist and hip circumferences, along with height, were measured as part of the main Tromsø 6 study at a median (interquartile range) of 5.2 (3.0–6.2) months before the RENIS-T6 investigations. Body weight was measured in the RENIS-T6 study to the nearest 0.1 kg on a SECA digital scale (SECA, Hamburg, Germany). The same weight scale was used for all subjects and was calibrated just before the study began. Height was measured to the nearest centimeter with a wall-mounted measuring tape. BMI was defined as height in meters divided by weight in kilograms squared. Waist and hip circumferences were measured horizontally over the umbilicus after exhalation and at the greatest protrusion of the buttocks, respectively. The WHR was calculated as the waist circumference divided by the hip circumference.

Subjects were classified into overweight and obesity categories based on cut-off values used by the World Health Organization and the International Diabetes Federation for European populations. BMI classes of 18.5–24.9, 25.0–29.9 and ≥ 30.0 define normal weight, overweight and obesity, respectively. WC categories of > 94 cm for men and > 80 cm for women represent “increased risk of metabolic complications”, while a WC of > 102 cm for men or > 88 cm for women, or a WHR of ≥ 0.90 for men or ≥ 0.85 for women represents “substantially increased risk” [38].

There were only four subjects with BMI <18.5, these were grouped with the normal BMI (18.5–24.9) group for the purposes of this study. Fifty-seven subjects had BMI between 35.0 and 39.9, and 5 subjects had BMI ≥ 40.0, these were included in the BMI ≥ 30.0 group.

### Definitions of hyperfiltration

The dichotomous variables for hyperfiltration were defined as unadjusted (absolute) GFR (mL/min) above the 90th percentile. We used two alternative definitions where the 90th percentile was either age-/sex- and height-specific (RHF_Height_) or age-/sex-/height and weight-specific (RHF_Weight/height_) (Table 1).

**Table 1:** Alternative definitions of renal hyperfiltration based on different adjustment variables in multiple linear regression

RHF definition	Dependent variable	Independent variables	Definition of dichotomous RHF variable
RHF_Height_	Logarithm of absolute GFR (in mL/min)	Sex and logarithms of height and age	Residual > 90th percentile
RHF_Weight/height_	Logarithm of absolute GFR (in mL/min)	Sex and logarithms of weight, height and age	Residual > 90th percentile

In both definitions, renal hyperfiltration was defined as residual > 90th percentile in multiple linear regression analysis with the independent variables listed above

*RHF* Renal hyperfiltration, *GFR* Glomerular filtration rate

In both cases, the respective 90th percentiles were calculated from multiple linear regression models, with the natural logarithm (ln) of unadjusted GFR (mL/min) as the dependent variable. For RHF_Height_, sex, ln(age) and ln(height) were used as independent variables, and for RHF_Weight/height_ ln(body weight) was added (Additional file 1: Table S1). A subject was defined as having RHF_Height_ or RHF_Weight/height_ if her regression residual was greater than the 90th percentile in the distribution of residuals in the regression analyses for the respective RHF definition (Table 1). This implies that the GFR cut-off for RHF for each individual depended on sex, age and height (RHF_Height_) or sex, age, height and body weight (RHF_Weight/height_). As an illustration, the GFR cut-off points for RHF in a male and female study participant with average measurements of age, height and weight are shown in Additional file 1: Table S2.

### Statistical analysis

The characteristics of the study population were tabulated as the mean (standard deviation) or median (interquartile range) for variables with skewed distributions. Pearson’s χ^2^ test, Welch’s *t*-test and the Mann-Whitney *U* test were used to calculate p-values for differences between the WHR groups, classified by the World Health Organization cut-off for WHR.

Separate multiple logistic regression analyses were performed with each of the two alternative RHF variables (Table 1) as the dependent dichotomous variable and categorical or continuous indices of obesity as the independent variable. Adjustments were made for age, sex, number of cigarettes smoked daily, ambulatory daytime systolic and diastolic BP and their interaction, and individual categories of antihypertensive medication (Model 1). Mathisen et al. found a statistically significant interaction between these BP variables and GFR in the same study population as the present study [37], which is why this interaction model was included. Model 2 included Model 1 and a dichotomous variable for a metabolically unhealthy lipid profile, defined as high-density lipoprotein cholesterol levels < 1.03 mmol/L in men or < 1.29 mmol/L in women, elevated triglyceride levels of ≥ 1.7 mmol/L, and/or use of lipid-lowering medication. The variables in Model 2 constitute two of the five established criteria used to define metabolic syndrome [39]. Model 3 included Model 1, fasting plasma glucose and insulin levels, and HOMA-IR. Model 4 included all models. Additionally, linear regression analyses using absolute and BSA-adjusted GFR as dependent variables and the same independent variables as above were performed.

Fractional polynomial regression analyses [40] were performed to see whether any obesity variables had non-linear relationships with either RHF variable or with mGFR as a continuous variable, adjusting for the same variables as in Model 4.

Statistical significance was set at *p* < 0.05. Statistical analysis was performed using STATA MP 14.0 software (www.stata.com).

## Results

### Study population

Thirty-three of the 1627 study subjects in the RENIS-T6 cohort were excluded due to undiagnosed diabetes mellitus. Another 39 subjects were excluded because of missing WC measurements, leaving 1555 subjects eligible for the current study (Fig. 1).

**Fig. 2:**
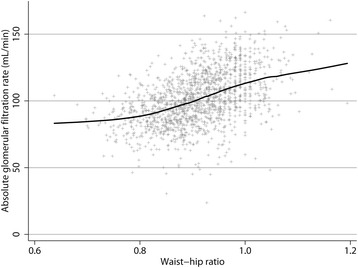
Scatterplot with locally weighted scatterplot smoothing (LOWESS) showing the relationship between the waist-hip ratio and glomerular filtration rate

**Fig. 3:**
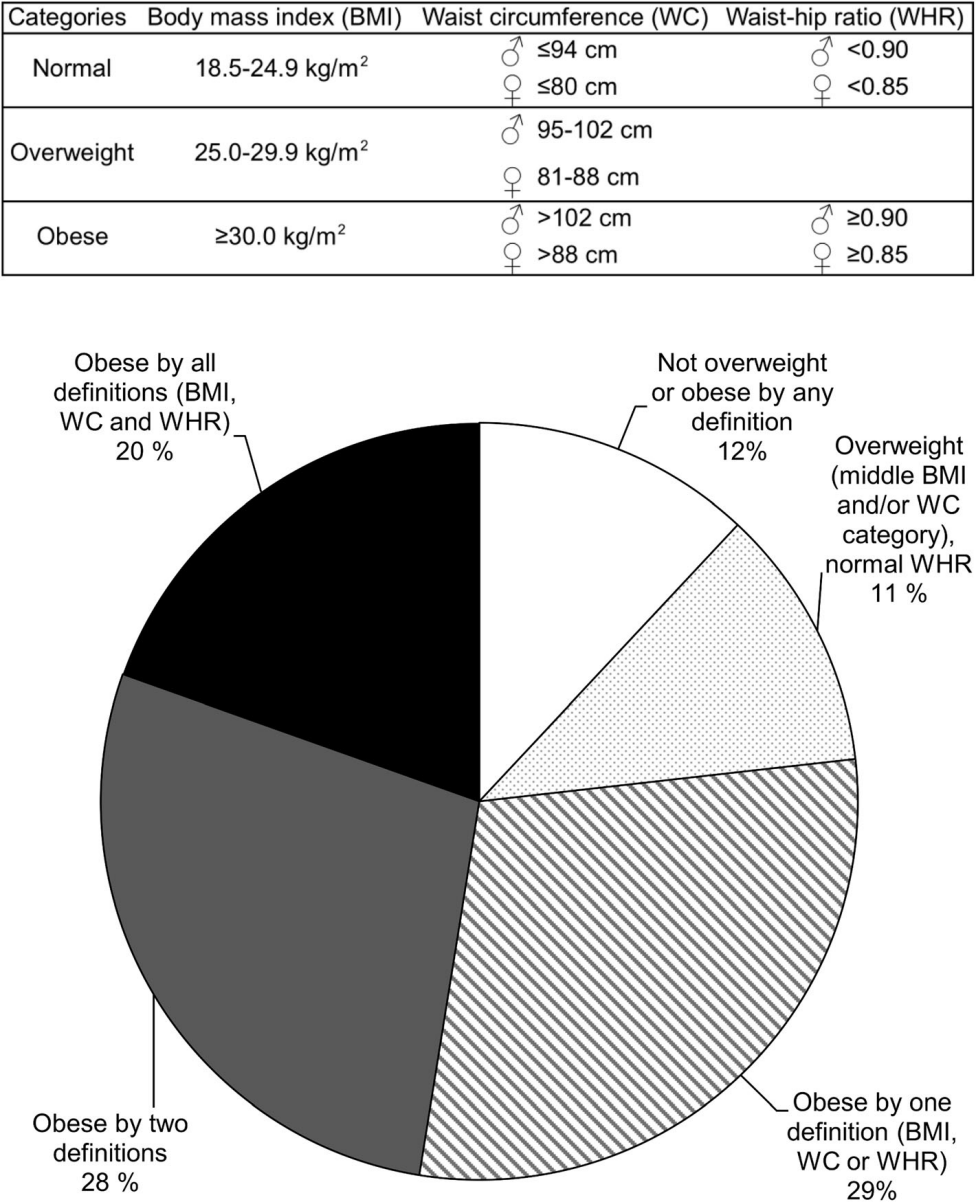
Distribution of obesity in the RENIS-T6 cohort, by WHO categories for body mass index (BMI), waist circumference (WC) and the waist-hip ratio (WHR)

The analysis of the study population showed several statistically significant associations between study variables and WHR categories (Table 2). A substantially higher percentage of males than females were obese according to the cut-off values. Subjects with a high WHR were, on average, older, had a higher absolute and BSA-adjusted GFR, higher BP, worse lipid and glucose profiles, and were more likely to use lipid- or BP-reducing drugs. There was a clear relationship between a greater WHR and higher GFR (Fig. 2). The vast majority of the population was overweight or obese (Fig. 3).

**Table 2:** Characteristics of the study population classified by World Health Organization waist-hip ratio cut-off point

	Normal waist-hip ratio^a^		Increased waist-hip ratio^b^		*P*-value
Subjects	432	27.8 %	1123	72.2 %	
Male gender	142	32.9 %	618	55.0 %	<0.001
Age	58.1	54.1–61.2	58.8	55.0–61.6	0.01
Waist-hip-ratio	0.824	0.046	0.941	0.055	
Waist circumference (cm)	83.5	7.6	99.3	9.7	<0.001
Body Mass Index (kg/m^2^)	24.6	3.0	28.3	3.8	<0.001
Height (cm)	168.9	8.5	171.3	8.8	<0.001
Weight (kg)	70.2	10.8	83.1	13.8	<0.001
Daily smokers	89	20.6 %	222	19.8 %	0.71
Daytime ambulatory systolic BP (mmHg)	126.1	12.6	131.5	13.0	<0.001
Daytime ambulatory diastolic BP (mmHg)	79.9	8.6	82.9	8.6	<0.001
Nighttime ambulatory systolic BP (mmHg)	108.5	12.2	111.9	12.2	<0.001
Nighttime ambulatory diastolic BP (mmHg)	64.9	8.6	67.0	8.4	<0.001
Office systolic BP (mmHg)	123.3	17.1	131.8	17.1	<0.001
Office diastolic BP (mmHg)	79.9	10.0	84.7	9.3	<0.001
Hypertension^c^	95	22.0 %	437	38.9 %	<0.001
ACE-inhibitor use	6	1.3 %	22	2.0 %	0.45
Angiotensin II-receptor blocker use	13	3.0 %	116	10.3 %	<0.001
Calcium-channel blocker use	7	1.6 %	71	6.3 %	<0.001
Beta-blocker use	7	1.6 %	60	5.3 %	0.001
Diuretica use	17	3.9 %	119	10.6 %	<0.001
Other anti-hypertensive medicine use	0	-	1	<0.1 %	0.54
Fasting glucose (mmol/L)	5.13	0.44	5.39	0.48	<0.001
Fasting insulin (mIU/L)	6.50	4.37–8.69	9.47	6.90–13.65	<0.001
HOMA-IR	1.47	0.98–2.01	2.30	1.60–3.37	<0.001
HbA1c (%)	5.46	0.30	5.57	0.34	<0.001
Cholesterol (mmol/L)	5.53	0.89	5.67	0.96	0.008
LDL cholesterol (mmol/L)	3.45	0.83	3.73	0.86	<0.001
HDL cholesterol (mmol/L)	1.75	0.44	1.45	0.39	<0.001
Triglycerides (mmol/L)	0.8	0.6–1.1	1.1	0.8–1.6	<0.001
Cholesterol-lowering drug use	21	4.9 %	79	7.0 %	0.12
Absolute GFR (ml/min)	93.8	16.0	104.0	20.4	<0.001
GFR (ml/min/1.73 m^2^)	90.1	13.0	92.0	14.8	0.02
RHFHeight	19	4.4 %	137	12.2 %	<0.001
RHFWeight/height	30	6.9 %	123	11.0 %	0.02

Data represented as number of subjects (percentage), median (interquartile range) or mean (standard deviation)

*BP* Blood pressure, *ACE* Angiotensin converting enzyme, *HOMA*-*IR* Homeostatic model assessment of insulin resistance, *LDL* Low density lipoprotein, *HDL* High density lipoprotein, *HbA1c* Hemoglobin A1c, *GFR* Glomerular filtration rate

^a^Female < 0.85, male < 0.90

^b^Female ≥ 0.85, male ≥ 0.90

^c^Office systolic BP ≥140 mmHg, office diastolic BP ≥90 mmHg and/or use of antihypertensive medication

### Hyperfiltration and obesity

The RHF definitions (Table 1) resulted in overlap, with 115 hyperfiltrating subjects having RHF by both definitions. Forty-one subjects had only RHF_Height_, while 38 had only RHF_Weight/height_.

In the logistic regression analyses, there was a statistically significant association between RHF_Height_ and all obesity variables, categorical and continuous, except for the intermediate WC category, even in the fully adjusted Model 4 (Table 3). This relationship remained significant when body weight was added to the regression analyses as an independent variable (Additional file 1: Table S3).

**Table 3:** Odds ratios for renal hyperfiltration using alternative renal hyperfiltration definitions and variable models

	Model 1			Model 2			Model 3			Model 4		
	OR	CI	*P*	OR	CI	*P*	OR	CI	*P*	OR	CI	*P*
RHF_Height_												
BMI < 25 kg/m^2^	1.00	(ref)		1.00	(ref)		1.00	(ref)		1.00	(ref)	
BMI 25–30 kg/m^2^	2.54	(1.45–4.47)	0.001	2.40	(1.36–4.24)	0.002	2.27	(1.28–4.05)	0.005	2.22	(1.24–3.95)	0.007
BMI > 30 kg/m^2^	8.03	(4.50–14.33)	<0.001	7.19	(3.99–12.94)	<0.001	6.11	(3.27–11.44)	<0.001	5.85	(3.12–10.99)	<0.001
BMI per 5 kg/m^2a^	2.66	(2.13–3.32)	<0.001	2.54	(2.02–3.19)	<0.001	2.40	(1.87–3.09)	<0.001	2.35	(1.83–3.03)	<0.001
WC < 80/94 cm	1.00	(ref)		1.00	(ref)		1.00	(ref)		1.00	(ref)	
WC 80–88/94–102 cm	1.89	(0.93–3.85)	0.08	1.82	(0.89–3.72)	0.10	1.63	(0.79–3.35)	0.18	1.62	(0.79–3.32)	0.19
WC > 88/102 cm	4.96	(2.59–9.49)	<0.001	4.48	(2.32–8.62)	<0.001	3.64	(1.86–7.14)	<0.001	3.52	(1.79–6.91)	<0.001
WC per 10 cm^a^	1.99	(1.68–2.35)	<0.001	1.92	(1.62–2.27)	<0.001	1.80	(1.50–2.17)	<0.001	1.78	(1.47–2.14)	<0.001
WHR < 0,85/0,90	1.00	(ref)		1.00	(ref)		1.00	(ref)		1.00	(ref)	
WHR > 0,85/0,90	2.91	(1.75–4.83)	<0.001	2.66	(1.59–4.43)	<0.001	2.24	(1.33–3.78)	0.002	2.17	(1.28–3.66)	0.004
WHR per 0.10^a^	2.67	(1.98–3.60)	<0.001	2.49	(1.84–3.37)	<0.001	2.20	(1.60–3.02)	<0.001	2.14	(1.55–2.94)	<0.001
RHF_Weight/height_												
BMI < 25 kg/m^2^	1.00	(ref)		1.00	(ref)		1.00	(ref)		1.00	(ref)	
BMI 25–30 kg/m^2^	0.84	(0.55–1.28)	0.42	0.79	(0.52–1.21)	0.28	0.72	(0.47–1.12)	0.14	0.70	(0.45–1.09)	0.11
BMI > 30 kg/m^2^	1.17	(0.72–1.90)	0.53	1.04	(0.63–1.71)	0.88	0.84	(0.49–1.45)	0.53	0.80	(0.46–1.39)	0.43
BMI per 5 kg/m^2a^	1.14	(0.92–1.42)	0.24	1.08	(0.86–1.36)	0.50	0.97	(0.75–1.26)	0.82	0.95	(0.73–1.23)	0.70
WC < 80/94 cm	1.00	(ref)		1.00	(ref)		1.00	(ref)		1.00	(ref)	
WC 80–88/94–102 cm	1.23	(0.72–2.10)	0.44	1.20	(0.70–2.04)	0.50	1.12	(0.65–1.92)	0.69	1.11	(0.65–1.91)	0.70
WC > 88/102 cm	1.56	(0.95–2.56)	0.08	1.44	(0.87–2.38)	0.16	1.29	(0.76–2.19)	0.35	1.25	(0.73–2.13)	0.41
WC per 10 cm^a^	1.21	(1.03–1.42)	0.02	1.17	(0.99–1.38)	0.07	1.11	(0.92–1.33)	0.28	1.09	(0.91–1.31)	0.35
WHR < 0,85/0,90	1.00	(ref)		1.00	(ref)		1.00	(ref)		1.00	(ref)	
WHR > 0,85/0,90	1.66	(1.07–2.55)	0.02	1.57	(1.01–2.42)	0.04	1.45	(0.93–2.27)	0.10	1.42	(0.90–2.22)	0.13
WHR per 0.10^a^	1.66	(1.24–2.21)	<0.001	1.59	(1.18–2.13)	0.002	1.51	(1.11–2.06)	0.009	1.48	(1.08–2.02)	0.01

*RHF* Renal hyperfiltration, *OR* Odds ratio, *CI* Confidence interval, *BMI* Body mass index, *WC* Waist circumference, *WHR* Waist-hip ratio

Model 1: Adjustment for age, sex, number of cigarettes smoked daily, ambulatory daytime systolic and diastolic BP (and their interaction), and individual categories of antihypertensive medication

Model 2: Model 1 and a dichotomous variable for a metabolically unhealthy lipid profile, defined as HDL-cholesterol levels < 1.03 mmol/L in men or < 1.29 mmol/L in women, elevated triglyceride levels of ≥ 1.7 mmol/L, and/or use of lipid-lowering medication

Model 3: Model 1 plus fasting plasma glucose and insulin levels, and HOMA-IR

Model 4: All models combined

^a^continuous variable

With RHF_Weight/height_, these relationships changed. Only the WHR as a continuous variable was consistently associated with RHF_Weight/height_ across all the models (*p* < 0.05). In Model 1, the odds ratio (confidence interval) for RHF_Weight/height_ was 1.66 (1.24–2.21) for each 0.10 increase in the WHR. The association was attenuated, but remained significant, when metabolic risk factors were added as independent variables in Models 2, 3 and 4 (Table 3).

Linear regression analyses with absolute and BSA-adjusted GFR as dependent variables and the same independent variables as above showed significant positive relationships between body size variables and absolute GFR, but no statistically significant relationship with BSA-adjusted GFR (Additional file 1: Table S4).

Interaction analyses were performed on the obesity variables and sex as well as the obesity variables and the dichotomous variable for an unhealthy lipid profile (defined in Model 2); but no statistically significant interactions were found. No statistically significant non-linear relationship was found between any obesity variables and the RHF variables or mGFR when analyzed in fractional polynomial regression models.

## Discussion

In this study of non-diabetic, middle-aged subjects from the general population, higher WHR, but not BMI or WC, was consistently associated with RHF, regardless of the RHF definition used. This finding suggests that excessive abdominal fat stores, as opposed to excess body weight distributed more evenly in the body, may potentially be more harmful to kidney function.

Most previous RHF studies with mGFR have found a positive relationship between BMI and RHF that disappears upon the adjustment of GFR to BSA [17–20]. The indexing of GFR to 1.73 m^2^ of BSA may be problematic in itself, particularly in the abnormal body sizes encountered when studying obese subjects [41]. Kwakernaak et al. found that the WHR predicted a lower BSA-adjusted mGFR when adjusted for BMI, age, sex and BP [18]. However, the sample size was small and consisted of kidney donors and volunteers, who may not be representative of the general population. Pinto-Sietsma et al. made a similar finding of higher WHR associated with lower GFR in a larger population, but the result was based on GFR estimated by creatinine clearance [22].

The hypothesis of hyperfiltration as a precursor to overt CKD, originally proposed by Brenner, is based on hyperfiltration in individual glomeruli [10]. Because it is not possible to measure single-nephron GFR directly in living humans, an indirect measure of hyperfiltration based on whole-kidney GFR must be used in epidemiological studies. Whole-kidney GFR is a function of single-nephron GFR and the total number of nephrons. Nephron numbers vary by gender and birth weight and decrease with age [42], and adult height has been shown to correlate with birth weight [43]. Thus, gender, height and age were included in both the RHF definitions (Table 1). RHF_Height_ used the age-, sex- and height-specific 90th percentile, and because an individual’s normal body weight is correlated with height, it provides an indirect adjustment for a theoretical “normal” body size. RHF_Height_ is thus defined as excessive GFR relative to the mean GFR for a person with “normal” body weight. Because GFR increases with increasing body weight and increasing metabolic needs [44], it follows that RHF_Height_ is associated with measures of obesity, as shown in Table 3. However, when body weight was added as an independent variable to the same RHF_Height_ logistic regression models as in Table 3, the results were attenuated but remained essentially similar (Additional file 1: Table S3), indicating that an obese figure is associated with hyperfiltration independently of the effect of weight itself.

Another way to correct for interindividual variation in weight is to include weight in the definition of hyperfiltration, as in RHF_Weight/height_. RHF_Weight/height_ accordingly defines hyperfiltration as excessive GFR relative to the mean GFR for persons with a given height and weight, whether obese or not. This definition may underestimate hyperfiltration in obese subjects, and RHF_Weight/height_ can be viewed as more conservative than RHF_Height_. The association of WHR with hyperfiltration even when using RHF_Weight/height_ is a strong indicator that central obesity also entails hyperfiltration at the glomerular level.

The merits of different body size measurement methods in the context of epidemiological research and risk estimates for disease have been debated, as have the merits of various cut-off points [38]. BMI has become the dominant measure of obesity, partly due to its well-established association with several obesity-related diseases and partly due to the near-universal availability of height and weight as variables in both large population studies and general clinical practice. WHR, which measures body fat distribution rather than absolute body size, has been shown to be at least equal to, and often better than, BMI as a predictor for obesity-related disease including CKD [22, 45–47].

The mechanisms of the adverse renal effect of abdominal adiposity are not fully understood, but some effects are known. The most severe and well-established mediators are increased risks of diabetes mellitus, hypertension and dyslipidemia [48–50]. The effects of metabolic risk factors can be observed in our results, with a gradual attenuation of the odds ratio for RHF when variables for an unhealthy lipid profile and insulin resistance were included in the regression analyses.

Additionally, some other mechanisms are known, including dysfunction in the renin-angiotensin-aldosterone system, increased tubular sodium reabsorption, and the effects of obesity-related hormones and cytokines such as leptin, adiponectin and Tumor Necrosis Factor-α [48–50].

Weight loss interventions, especially bariatric surgery, have been shown to reduce GFR in hyperfiltrating obese subjects [51]. However, most studies of such interventions have been small, and few studies have been published on long-term effects beyond the first 2 years after the interventions. A recent study by Zingerman et al. suggested a possible reversal of RHF in obese patients using acetazolamide, although the study did not include a placebo arm [52].

The strength of the present study lies in the measurement of GFR with a gold-standard method in a large, representative, mostly healthy cohort in an age group susceptible to early stages of chronic diseases. To our knowledge, this is the largest cohort from the general population that has been studied using precise GFR measurements. The exclusion of subjects with diabetes, cardiovascular disease and renal disease from the study population allowed us to focus on the preliminary stages of potential future CKD with less confounding from these high-risk patient groups. These groups would have been more likely to have passed the transient stage of hyperfiltration into a state of normal-range GFR, perhaps accompanied by slight albuminuria.

There are several limitations to this study. First, it was a cross-sectional study and thus could not prove causation, only correlation. Second, the study population was exclusively Caucasian and middle-aged, which may limit the transferability of findings to other population groups. Furthermore, while GFR was measured with a gold standard method, obesity was measured indirectly with anthropometric data, and not directly with gold standard dual energy X-ray absorptiometry, computed tomography or magnetic resonance imaging methods. Glucose and HbA1c were only measured once to exclude diabetes, while regular clinical practice requires two measurements for the diagnosis.

## Conclusions

We conclude that the WHR is associated with RHF, independently of other risk factors and even using RHF_Weight/height,_ a conservative, body size-adjusted RHF definition. Longitudinal studies are needed to explore whether RHF predicts future non-diabetic CKD. Further studies on whether the WHR predicts CKD better than other obesity measurements are also warranted.

## Additional file

Additional file 1: Table S1. Regression models for the alternative renal hyperfiltration definitions. **Table S2.** mGFR cut-off points for renal hyperfiltration in male and female subjects from the study cohort with average height, weight and age. **Table S3.** Odds ratio for RHF_height_, with body weight added as an independent variable. **Table S4.** Multiple linear regression with measured GFR and continuous obesity variables. (XLSX 22 kb)

## Abbreviations

BMI: Body mass index
BP: Blood pressure
BSA: Body surface area
CKD: Chronic kidney disease
ESRD: End-stage renal disease
GFR: Glomerular filtration rate
HOMA-IR: Homeostatic model assessment of insulin resistance
mGFR: Measured glomerular filtration rate
RENIS-T6: Renal Iohexol Survey in Tromsø 6
RHF: Renal hyperfiltration
WC: Waist circumference
WHR: Waist-hip ratio
